# Improving malaria chemoprevention coverage in pregnancy: Surveying stakeholder preferences for new product profiles and community-delivery approaches across five African countries

**DOI:** 10.1371/journal.pgph.0005607

**Published:** 2026-03-13

**Authors:** Céline Audibert, Isabelle Borghini-Fuhrer, Myriam El Gaaloul, Hanu Ramachandruni, André-Marie Tchouatieu, Maud Majeres Lugand

**Affiliations:** MMV Medicines for Malaria Venture, Geneva, Switzerland; Malaria Research and Training Center, University of Science, Techniques and Technology of Bamako, MALI

## Abstract

Sulfadoxine-pyrimethamine (SP) is the only WHO-recommended option for the intermittent preventive treatment of malaria in pregnancy (IPTp). However, coverage is suboptimal, efficacy is limited in areas of high resistance and SP is contraindicated in the first trimester and women living with HIV receiving cotrimoxazole. This study investigated stakeholder perspectives on next-generation IPTp product characteristics and their potential impact on uptake, adherence and community deployment. Surveys were conducted in five malaria endemic countries, including pregnant women (n = 75), nurses/community health workers (CHWs) (n = 53), clinicians (n = 75) and policymakers (n = 51). Surveys included quantitative elements and open-ended questions to examine stakeholder perceptions of chemoprevention product characteristics influencing uptake, adherence and implementation. Additionally, a discrete choice experiment (DCE) conducted among clinicians and policymakers compared next-generation IPTp oral product profiles against SP. Across stakeholders the most important attribute was safety in all trimesters, followed by short duration of therapy, few tablets per day, food requirements and impact on birthweight was the least influential. Clinicians and policymakers also believed resistance markers to be important. All stakeholder groups indicated that a long-acting injectable with at least three months’ coverage would enhance adherence. For oral medication, pregnant women believed being able to take food with treatment was the most important attribute to enhance adherence, while a four-tablet daily dosing would decrease adherence. Clinicians and policymakers felt that delivery by CHWs would increase adherence and expand IPTp uptake in currently unreached women. However, three-quarters of pregnant women preferred ante-natal clinic delivery of a new medicine. The DCE favored products without food restrictions most strongly. This study offers important insights for the development and deployment of next-generation IPTp products. Minimizing food-related restrictions and addressing resistance concerns are key priorities, together with safety and tolerability. Long-acting injectable formulations and hybrid delivery models could play a substantial role in improving adherence and uptake.

## Introduction

In 2024, malaria infection affected an estimated 13 million pregnant women across African countries with moderate-to-high malaria transmission – around a third of all pregnancies [1]. Pregnant women face a heightened risk of malaria infection compared to their non-pregnant counterparts, along with an increased likelihood of adverse outcomes, such as severe maternal anemia, placental malaria, severe malaria and maternal mortality [2,3]. Furthermore, malaria during pregnancy poses significant risks to the fetus and newborn, including intrauterine growth restriction, miscarriage, stillbirth, premature delivery and low birthweight – a key risk factor for infant mortality [2–4].

The World Health Organization (WHO) advises a comprehensive strategy for managing malaria during pregnancy, including the use of insecticide-treated bed nets for prevention and timely and effective case management. In addition, a key intervention in regions of moderate-to-high malaria transmission in Africa is the intermittent preventive treatment of malaria in pregnancy (IPTp) with sulfadoxine-pyrimethamine (SP) (IPTp-SP) [5]. SP is administered at monthly intervals starting from week 13, regardless of the pregnant woman’s malaria infection status, with the aim of achieving at least three doses (IPT3+). This is usually delivered via routine ante-natal care (ANC) visits with directly observed therapy (DOT) [5].

IPTp-SP was first recommended by the WHO in 1998 [6], but uptake has been limited. In 2023, across 34 African countries only 44% of eligible pregnant women received the recommended three or more SP doses, well below the WHO target of 80% coverage [1]. Recently, the WHO published guidance on community-based IPTp-SP (c-IPTp), with the aim of increasing coverage via established networks of community health workers (CHWs) [7]. Even so, additional barriers to access and acceptability, such as the fear of adverse events, lack of knowledge and misperception, inadequate trust in CHWs, sociocultural factors and healthcare system challenges, such as stock outs and staff shortages, will need to be addressed to ensure more women are protected [8,9].

There are key pharmacological limitations with SP for malaria chemoprevention. While pregnant women are most at risk of malaria infection and its complications during the first trimester [10–13], safety and tolerability of SP in the first trimester of pregnancy has not been established [5]. The co-occurrence of HIV and malaria increases the risk of placental damage and adverse pregnancy outcomes [14]. Treatment with the antibacterial combination of the antifolates sulfamethoxazole and trimethoprim (Cotrimoxazole) has direct benefits to the mother and significantly improves birth outcomes in patients co-infected with HIV [15,16], but SP is contraindicated for use with cotrimoxazole due to drug–drug interactions [5]. Finally, pregnant women with sulfonamide allergies are ineligible for any SP treatment. These gaps in chemoprotection underline the historical neglect in developing antimalarial drugs for use during pregnancy [17]. Importantly, there are concerns regarding the emergence and proliferation of *Plasmodium falciparum* high-level resistance to SP [18], causing SP to be no longer recommended as a treatment for uncomplicated malaria. In addition, the presence of high resistance to SP (Lys540Glu) reduces the efficacy of IPTp with SP by over three-fold [18,19], although its effectiveness in reducing the risk of low birthweight appears mostly intact [9]. New solutions for preventing malaria in pregnancy and which improve uptake and acceptability are urgently required [20].

The development of new combinations of approved antimalarial drugs into new chemoprevention products is an accelerated path to new products, as much of the preclinical data, clinical safety and antimalarial efficacy are already available. This approach was included in the WHO preferred product characteristics for malaria chemoprevention [21]. Pyronaridine (PYN) and piperaquine (PQP) are components of the artemisinin-based combination therapies (ACTs) artesunate-pyronaridine (ASPY) and dihydroartemisinin-piperaquine (DHA-PQP), approved for the treatment of uncomplicated malaria [5]. Based on their known pharmacological characteristics, a novel combination of PYN and PQP has been considered for chemoprevention [21]. Both drugs possess long half-lives which are suitable for once monthly dosing [22–24]. They appear to have distinct mechanisms of resistance, although the precise molecular mechanisms of resistance have not been fully elucidated [25,26]. Neither drug has demonstrated teratogenic effects [27,28], indicating potential for use during all trimesters of pregnancy [29,30]. Furthermore, no significant overlaps in the known adverse event profiles of PYN and PQP have been identified that would prevent their combined use [27,28]. In a phase I study evaluating PYN and PQP alone and in combination, there were no clinically concerning safety signals and the combination was well tolerated in healthy volunteers [31]. Potential disadvantages are that PQP exposures are increased as much as three-fold with food [31], and ASPY and DHA-PQP are given once daily for three days, whereas SP is given on a single day. However, there may be opportunities to adjust the PYN-PQP regimen specifically for chemoprevention. PYN-PQP would offer key potential advantages over SP including compatibility with cotrimoxazole, suitability for women with sulfonamide allergies and expected good tolerability throughout pregnancy. However, the final product profile for PYN-PQP is yet to be determined.

Although the pharmacology, safety and efficacy of new chemopreventive products are conventionally evaluated through clinical studies, their potential impact on the communities and contexts in which they will be deployed is rarely considered. To develop a product that best addresses the needs of the communities it is intended to benefit, this research aimed to explore stakeholder preferences for IPTp intervention in five malaria-endemic countries. As well as investigating preferences for specific possible PYN-PQP product profiles, and the potential impact of IPTp product characteristics on uptake and adherence, the study also evaluated the opportunities for community deployment to support new product introduction. These insights can guide product development by ensuring that formulations, dosing regimens and implementation strategies are tailored to maximize acceptability and effectiveness in real-world settings.

## Materials and methods

### Ethics statement

The William Davidson Institute (WDI) at the University of Michigan submitted a research plan for this study to the University of Michigan Institutional Review Board (IRB) for Human Subjects, which determined the study was exempt from IRB review given its non-interventional nature. For country-level approvals, supporting letters were obtained from the Ministry of Health National Malaria Programs in the Democratic Republic of Congo, Nigeria, Uganda and Kenya. Ghana provided specific clearance from their National Ethics Review Board (Ghana Health Service Ethics Review Committee: 004/04/24). All participants were informed of the purpose of the study and provided written consent for use of their de-identified data, analysis and publication. All data were stored securely.

### Study context

The research was conducted between April 24 to September 7, 2024, across five African countries. Democratic Republic of Congo (DRC): Boma, Kenge, Kinshasa (10 May to 26 June); Ghana: Accra, Bekwai, Kanvilli, Kumasi, Obuasi, Savelugu, Tamale, Tema, Vittin, Yendi (2 July to 25 August); Kenya: Bondo, Bungoma, Busia, Homabay, Kakamega, Kilifi, Kisumu, Malingi, Mombasa, Mwatate, Nairobi, Siaya, Voi (28 May to 7 September); Nigeria: Abuja, Benin, Kano, Lagos, Port Harcourt (24 April to 20 June); and Uganda: Iganga, Jinja, Kampala (7 June to 30 June).

Together, these five countries account for over 50% of global malaria cases and deaths, with DRC, Ghana, Nigeria and Uganda being designated ‘high burden to high impact’ countries [1]. The study aimed to capture as much diversity as possible, while maintaining relevance. The study countries encompassed West Africa (Ghana, Nigeria), Central Africa (DRC) and East Africa (Kenya, Uganda) providing geographic and social diversity [1]. Also, the prevalence of high-level resistance to SP varied across the countries, being highest in in East Africa [32–34]. Each country has established IPTp programs, though uptake varies, with only DRC, Ghana and Uganda achieving >50% IPT3+ in 2024 [1]. Additionally, c-IPTp had been previously piloted in DRC and Nigeria as part of the Transforming IPTp for Optimal Pregnancy (TIPTOP) project [35].

Study sites were selected across a mix of urban and rural settings in malaria endemic regions based on the current implementation of IPTp-SP and malaria transmission. Participants were chosen to span all aspects of the intervention across four key stakeholder groups – pregnant women, nurses/CHWs, clinicians and policymakers. Since CHWs have differing roles, situations within the healthcare system and remuneration/incentivization arrangements across countries, they were defined broadly in this study as individuals without professional healthcare qualifications involved in the delivery of maternal healthcare interventions in the community.

### Study design

The study investigated different aspects of IPTp across three domains: perceptions of chemoprevention product characteristics; potential impact of product characteristics on adherence; and the potential for c-IPTp on increasing intervention coverage. Surveys were used to assess stakeholder perspectives, including both quantitative elements and open-ended questions. For product characteristics, survey findings were amalgamated across countries, as chemoprevention products will not be tailored to country-specific preferences. However, For c-IPTp, the survey findings by country were considered as they may impact uptake and thereby market penetration of new products. Additionally, preferred product profiles for next-generation IPTp versus SP were evaluated among clinicians and policymakers via a direct choice experiment (DCE). The DCE allowed identification of country-specific product preferences which could potentially limit the geographic utility of new chemoprevention products.

### Participants

Each of the four stakeholder groups were recruited using specific inclusion criteria (Table 1). Participants were identified and interviewed by a field partner (Sanisphere, Abuja, Nigeria).

**Table 1:** Inclusion criteria for each category of participant.

Category	Sub-category	Inclusion criteria
Pregnant women	–	Women currently pregnant or pregnant in the last 12 months
Nurse/CHW	Nurse	At least 2 years professional experience AND Experience of providing health services to pregnant women
	CHW	
Clinicians	General practitioner	At least 5 years of professional experience AND Experience with IPTp-SP
	Gynecologist	
	Health facility manager	
Policy makers	–	National Malaria Program representative or in-country implementing partner with at least 5 years of professional experience AND Experience of malaria prevention or treatment AND Experience with IPTp-SP

CHW, community health worker; IPTp-SP, intermittent preventive treatment of malaria in pregnancy with sulfadoxine-pyrimethamine.

A convenience sampling approach was used. To minimize selection bias, the target population and research questions were clearly defined, and participants were recruited through multiple sources across different settings to ensure diversity and representativeness. For policymakers, a first cohort was screened from existing contacts, with these providing additional contacts, and so on until target sample size was achieved. For clinicians, interviews started with health center managers who identified the most relevant clinicians among the staff. Physicians and health center managers guided the selection of nurses and CHWs. Pregnant women were identified through health providers among patients who were attending care during the data collection timeframes.

Target sample size was 75 pregnant women, 75 clinicians, 50 nurses/CHWs and 50 policymakers for a total target of 250 participants evenly distributed across the five countries. The sample size was chosen for feasibility while capturing diverse insights from all stakeholder groups and countries. Demographic information was collected to assess sample characteristics and potential recruitment imbalances. Thematic saturation was not a requirement for sampling as the objective was to capture the main drivers of decision making and behavior around IPTp based on stakeholder priorities for new chemoprevention products, rather than to provide an exhaustive analysis of opinion around IPTp.

### Survey development

A bespoke survey examining the research questions and incorporating both quantitative and qualitative elements was developed for each stakeholder group by the authors and WDI. Likert scales were used to obtain quantitative data. Open-ended questions and discussion points generated qualitative responses. Survey questions were formulated based on a PubMed literature search for published studies considering IPTp implementation and WHO recommended strategies, including community-based deployment of IPTp [5,7]. The resulting survey questionnaires and the DCE were piloted in Nigeria with five respondents, including one patient, one nurse, two clinicians and one policymaker. A debrief workshop was organized with the researchers, field partner representatives and interviewers to refine the questionnaires to improve the interview flow and facilitate information collection.

### Data collection procedures

Demographic, survey and DCE data were collected by the field partner via face-to-face interviews. All instruments were applied on an individual basis by native speakers who had no prior relationship to participants. Interviewers had healthcare backgrounds, with at least a master’s degree in pharmacy, were professionally employed as market researchers, and received specific training regarding the study scope, purpose and interview conduct. All interviews for the survey and DCE, including audio recordings, were captured directly into the online survey platform Alchemer (version 6.5, Louisville, CO, USA). Verbatim transcriptions were translated into English with the veracity of translation confirmed by native language speakers. Interviews took place in health facilities and lasted around 30–45 minutes for pregnant women, 45–60 minutes for nurses/CHWs and 60–90 minutes for clinicians and policymakers. Data were de-identified before analysis.

### Survey data analysis

Analysis was conducted by WDI under the guidance of CA and MML. Quantitative analysis was performed in Python (https://www.python.org/) using descriptive statistics. For open-ended question data, the research team performed thematic analysis using NVivo (version 14, Denver, CO, USA) to systematically code and categorize data. The process involved initial familiarization with the data, generation of initial codes, development of a coding framework and iterative review and refinement of themes until consensus. The coding framework was developed through a combination of deductive and inductive approaches. Deductively, pre-defined codes were informed by the survey questions and the literature review. Inductively, new codes emerged from participants’ perspectives during open coding. The initial code list was drafted, with iterative discussion to achieve consensus. The resulting coding framework included over 100 finalized codes, capturing the breadth and depth of participant responses. Each response was coded objectively without inferring meaning beyond what was stated. Respondents may have provided a single reason within one response, multiple reasons (within one response), no response, or a response with no insight (which could not be coded). The frequency of similarly coded responses was calculated, and the top most frequent themes were reported (see supporting information).

The structure of the survey, including both quantitative and qualitative components, allowed triangulation of responses. Using Likert scales, respondents could indicate the importance of a particular factor either in the positive or negative directions. Thus, after coding, those who believed a particular characteristic was of high importance could be divided among those who thought a particular characteristic was beneficial and those who believed it to be detrimental. Also, coding captured the reasons why respondents believed characteristics were of lower importance or neutral opinions. Illustrative quotes were selected to demonstrate why attributes were considered i) important in a positive way; ii) important in a negative way; iii) not as important as other factors. In this way the diversity of opinion could be represented.

### Discreet choice experiment

The DCE examined policymakers’ and clinicians’ preferences for specific oral next-generation IPTp product profiles versus SP. This method assumes that individuals rationally chose a product with the greatest benefit by accepting trade-offs between different characteristics [36]. This is not comparable with the survey approach which allows open preferences rather than closed choices. The DCE was only completed by policymakers and clinicians due to its time-consuming and technical nature, requiring a comprehensive understanding of various competing product characteristics, demanding knowledge that extends beyond personal experience.

The authors and WDI generated the DCE model. DCE design and analysis were performed in JMP (version 18 Cary, NC, USA) using a Panel Mixed Logit model in the JMP Consumer Research module. Six key product characteristics were evaluated to cover potential differences between products in convenience, efficacy, safety and resistance profiles (Table 2). The characteristics and their options were designed to simulate the standard-of-care (SP) and different potential profiles for a next-generation product (PYN-PQP). Each participant reviewed nine profile pairs. Results from the DCE were used to estimate the probability of selection of a new product such as PYN-PQP versus SP. Four separate PYN-PQP profiles were simulated, each representing a different combination of anticipated characteristics of an orally administered product. Marginal utility values were used to estimate the relative importance of each characteristic and potential PYN-PQP profiles were ranked based on the probability of selection [37]. Preference shares for the 216 combinations of the next-generation IPTp products over SP were determined. Product characteristics with a significance level <0.05 were considered statistically relevant to product choice.

**Table 2:** Product characteristics evaluated in the discreet choice experiment.

Product characteristics	Choice options
Days per course	1; 2; or 3 days
Tablets per day	2; 3; or 4 tablets
Safety in each trimester	safe to start in any trimester; safe to start in second or third trimester
food requirements	can be taken with or without food (no food requirement); must be taken with food (fed); must not eat 3 h before or 3 h after taking product (fasted)
impact on birthweight	increases average infant birthweight by 90 g; no impact on infant birthweight
resistance markers	presence of known resistance markers; absence of known resistance markers

## Results

### Participants

Overall, 254 participants were included across the five countries (Table 3). Participants’ characteristics by country are available in the supplementary information (S1 Table). Overall, 80% (60/75) of the ‘pregnant women’ category were currently pregnant, 100% (75/75) had attended at least one ANC visit, 95% (71/75) had received at least one dose of IPTp-SP, none were on cotrimoxazole, 39% (29/75) lived in rural areas, 27% (20/75) in peri-urban and 35% (26/75) in urban settings. Clinicians, nurses and CHWs were also distributed among rural (27% [35/128]), peri-urban (33% [42/128]) and urban (40% [51/128]) settings and were mainly employed in public health care settings or by the government (69% [75/108]), with most providing direct care to pregnant women (94% [108/115]). Most policymakers (94% [48/51]) had medium- or high-level responsibility for decisions about new antimalarial drugs, had been working in malaria for a median of 14.0 years (interquartile range 8.5–22.0) and in their current role for a median of 5.0 years (2.2–8.5).

**Table 3:** Number of participants by category, sub-category and country.

Category	Sub-category	Number of participants					
		DRC	Ghana	Kenya	Nigeria	Uganda	Total
Pregnant women	–	15	15	15	15	15	75
Nurse/CHW	Nurse^a^	10	7	7	7	7	38
	CHW	3	3	3	3	3	15
Clinicians	General practitioner	6	6	7	4	5	28
	Gynecologist	6	6	5	8	7	32
	Health facility manager	3	3	3	3	3	15
Policy makers	–	10	10	10	11	10	51
Total		53	50	50	51	50	254

CHW, community health worker; –, there were no sub-categories for pregnant women or policy makers.

^a^ Included a small number of midwives and clinical officers.

### Perceptions of malaria chemoprevention product characteristics

**Fig 1:**
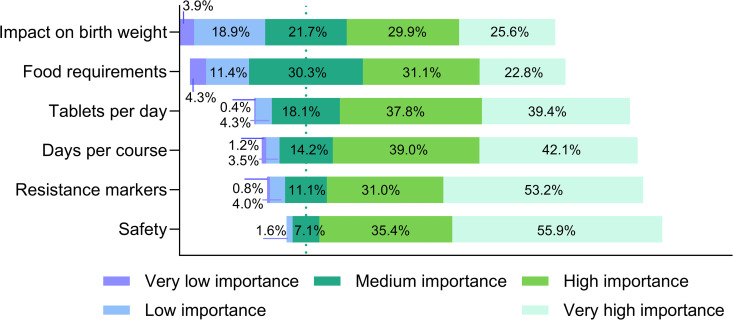
Importance of malaria chemoprevention product characteristics. All participants (n = 254) expressed preferences for product characteristics using a Likert scale, except for resistance markers, which was only considered in the surveys for clinicians and policymakers (n = 126).

Perceptions of individual product characteristics were examined. As a first step, a Likert scale was used across all participant categories to assess the importance of five chemoprevention product characteristics (safety, days per course, tablets per day, food requirements and impact on birthweight), as well as the importance of resistance markers among clinicians and policymakers only (Fig 1). The potential for co-administration with co-trimoxazole was also investigated with nurses, clinicians and policymakers. Safety, days per course and tablets per day all had similar levels of importance, whereas food requirements and impact on birthweight were considered of lower importance. The reasons behind participants’ responses on the Likert scale were further explored thematically, summarized below (S2 Table).

#### Safety.

For those respondents who rated safety as of very high or high importance, the most frequently mentioned reason was the desire for a drug which was safe to take in any trimester, unlike SP. The need to protect pregnant women from malaria was also highlighted. Some respondents were concerned that drugs may have a teratogenic effect during the period of organogenesis (i.e., the first trimester). This was also raised among those who rated safety of medium or low/very low importance, but who felt that safety was of secondary importance to other factors, such as adherence.

- *I think it is better if it can start in the first trimester as I have had malaria before in the beginning of my pregnancy and they had to treat me. (Pregnant woman)*
- *I wouldn’t want to give a drug in the first trimester because some women are very fragile and any little thing can affect the fetus. (Nurse/CHW)*
- *Regardless of the trimester the treatment starts, they have to be taken to completion. (Policymaker)*

#### Days per course.

Respondents who rated days per course as high/very high importance had concerns related to pregnant women’s adherence and underdosing due to forgetting to take the medicine. Aspects of pregnancy affecting uptake, such as feeling tired or nauseous, as well as women’s aversion to drugs were also considered important. Among other respondents, alternative factors were believed to be more critical, such as efficacy and tolerability, and there was opinion that any aversions to multiple days per course would be overcome by patient education emphasizing the benefits of the therapy.

- *When the number of days in a month is increased, patient may likely not comply, and this will lead to therapeutic failure and also drug resistance. (Nurse/CHW)*
- *I will still prefer something this simple as it will ensure compliance. Higher number of days per course will lead to a reduced compliance. (Policymaker)*
- *Increasing the number of days will not necessarily make me not take my medications once I know the importance of adherence to my medications. (Pregnant woman)*
- *It is not the number of days per course that a drug is taken that is important, it is the efficacy of the drug hence my choice of very low importance. (Clinician)*

#### Tablets per day.

The main concern among those who ranked tablets per day of high/very high importance was pill burden, given the large number of tablets, including drugs and supplements, which are administered during pregnancy. Also, taking pills while pregnant and potentially nauseous is a consideration. Some women were struggling to take three SP tablets, whereas others did not mind about the number of pills if they only had to take the pills once a month and the drug were effective, so there was an element of personal preference. There was again the suggestion that patient education could increase acceptance, and those who thought tablets per day was of low/very low importance believed efficacy, ensuring adherence through DOT and a once-a-day regimen to be more important.

- *I am struggling with three tablets already and you are suggesting four tablets, you obviously do not want me to be taking my medications. (Pregnant woman)*
- *It’s more of how effective the drug would be in protecting me from malaria than the number of tablets in a day. (Pregnant woman)*
- *The psychology of taking too many drugs will make them feel like they are sick which will in turn reduce compliance. (Clinician)*
- *It’s of importance, but not so much, as the patients will take the drugs depending on how well we make them know the importance of the medication. (Nurse/CHW)*

#### Food requirements.

Supportive arguments for food intake before taking the drug emphasized the reduction in adverse events and the importance of maintaining energy levels for pregnant women. The flexibility for women to eat without restriction was valued because eating routines are often disturbed by pregnancy. There was a concern that some women may not be able to access food when required, citing high cost and limited availability, as well as issues around food intake during pregnancy because of nausea/vomiting/appetite loss. Further categorization of the responses indicated that a similar proportion of pregnant women (41%; 31/75) and nurses/CHWs (43%; 23/53) preferred that the drug be taken with food, compared with only 20% (15/75) of clinicians and just 6% (3/51) of policymakers. Those who considered food requirements of low importance were more focused on efficacy and adherence to medication.

- *Even if the drug is to be taken with food, I also don’t mind because food is my friend in pregnancy. (Pregnant woman)*
- *I think there will be biases because not all of us have access to food at the same time so I don’t want a drug that has a food requirement. (Pregnant woman)*
- *Most of the pregnant women complain of vomiting and other side effects. I feel like if there is food in the stomach, it will reduce these side effects. (Nurse/CHW)*
- *Pregnancy demands a lot of meals and nutrition; it is not something you tell them; to fast because of a drug. I prefer something that will not interrupt their meals. (Clinician)*
- *When they feel there is a condition around their eating or not eating, they tend to shy away and this has a negative effect on our ability to carry out DOT. (Policymaker)*

#### Impact on birthweight.

The top reasons why birthweight was considered of high/very high importance included both positive and negative aspects. On the positive side, avoidance of low birthweight, particularly in mothers who had poor nutrition, and the association of higher birthweight with favorable newborn health were mentioned. On the negative side, participants were concerned about difficult delivery with a heavier baby, the risk of Cesarean section, and other risk factors associated with heavier babies, such as gestational diabetes. Those who rated this attribute of medium/low/very low importance were more concerned with the preventive efficacy of the medicine and/or considered a 90 g birthweight gain as insignificant. When pregnant women were specifically asked using a 5-point Likert scale their opinion on having a baby which was 90 g heavier than average birthweight, there were notable differences between countries, with good/very good responses varying from 87% (13/15) in Ghana, 87% (13/15) in Uganda 73% (11/15) in Kenya, 47% in DRC (7/15) to just 33% (5/15) in Nigeria, suggesting a local effect on perceptions.

- *If I give birth to a slightly heavier child that works well for me since it means the child is healthy. (Pregnant woman)*
- *A heavier baby means a healthy baby and the mothers will be happy. Moreover, big babies' chances of survival are high. (Policymaker)*
- *We do not want a situation where the baby becomes too big where it now leads to assisted vaginal delivery or Cesarean section. (Clinician)*
- *I do not think a drug increasing the birthweight by 90 g or not would affect the compliance of the medicine. So, it is of low importance to me. (Nurse/CHW)*

#### Resistance markers.

This criterion was only evaluated among policymakers and clinicians. Those who thought that this attribute was of high/very high importance emphasized the need to ensure that a drug has low resistance and is effective. Concerns around molecular markers of drug resistance were also mentioned, signaling the emergence and potential spread of resistant parasites, undermining efficacy. Respondents who thought resistance was of low/very low importance highlighted that SP is still efficacious despite the detection of molecular resistance markers and suggested that there were other priorities with a new medicine, given that resistance will take time to develop.

- *We don’t want a medication that we will give and the women will still come down with malaria. We are experiencing a little of it with SP now. (Clinician)*
- *Resistance should not be key priority right now. If the medication is that good, let’s first deploy it before we start getting concerns about resistance. (Policymaker)*

Policymakers and clinicians were also asked about their perceptions of using the same antimalarial molecules for chemoprevention that are also combined in ACT products for treatment. Responses were categorically coded, with 38% (49/126) of participants open to using PYN-PQP as chemoprevention, citing the advantages of using combination therapies. However, they emphasized that more research would be needed, including monitoring for the emergence of drug resistance and recognized that new molecules would be preferable. Half of the participants (52% [65/126]), however, had strong concerns. These were mainly around the potential for drug resistance, particularly the volume of PQP use, given that DHA-PQP is already used for treatment and for chemoprevention in some settings. There were also policy considerations, with existing guidelines aiming to protect ASPY and DHA-PQP efficacy by reserving all the components of these products for malaria treatment.

- *I think using them for prevention would be great, but since we are using them for treatment at the moment, it should be better we get other molecules for prevention. (Policymaker)*
- *Some patients are familiar with the piperaquine molecule, which will lead to misuse and, unfortunately, the development of resistance. (Clinician)*
- *We have a policy that once a drug is used for treatment it cannot be used for chemoprevention due to issues of resistance – so both technically and from a policy point of view this is not acceptable. (Policymaker)*

#### Cotrimozazole co-administration.

Clinicians and policymakers were asked to estimate the proportion of pregnant women living with HIV who were taking cotrimoxazole regularly. Although only a small proportion (1.3%) of pregnant women were estimated to take cotrimoxazole, 74% (120/162) of nurses, clinicians and policymakers were highly or very highly interested in a new malaria chemoprevention product that would be safe in this population, expressing a need for a product which all women could use, regardless of their preexisting conditions. However, some were skeptical of the effectiveness of cotrimoxazole or highlighted the increased pill burden of taking both co-trimoxazole and malaria chemoprevention.

- *Somehow not allowing them partake in IPTp-SP can seem as though they are being ‘stigmatized’, so giving them that opportunity to take what the other pregnant women are taking will help reduce this stigmatization. (Policymaker)*
- *Some pregnant women cannot tolerate cotrimoxazole. This product has many side effects: weakness, dizziness, choking, etc. (Nurse)*
- *I will prefer a single drug that gives both HIV and malaria prophylaxis to avoid the pill burden. (Nurse)*

### Impact on adherence

**Fig 2:**
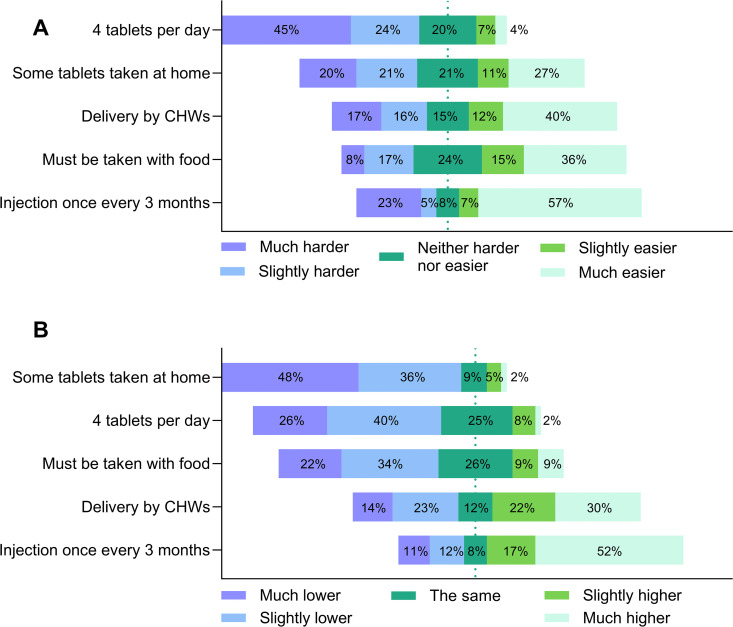
Impact of product characteristics on adherence. **A)** Quantitative assessment of the response of pregnant women (n = 75) to the question ‘with the proposed change, it would be [response] to take all doses. **B)** Quantitative assessment of the response of nurses/CHWs, clinicians and policymakers (n=179) to the question ‘With the proposed change, adherence would be [response].’.

The impact of different product characteristics and delivery by CHWs on adherence was investigated quantitatively using Likert scales with reference to current standard-of-care with SP (Fig 2). The attribute with the greatest expected positive effect on adherence across all participant categories was switching from a once-per-month oral medication to a once-per-three-months long-acting injectable. Pregnant women thought that having to take four tablets per day would be the most challenging to adherence (Fig 2A), whereas other stakeholders thought that taking some tablets at home would have the greatest negative impact on adherence (Fig 2B). These findings were further explored thematically with pregnant women and nurses/CHWs (discussed below), as these stakeholders have the greatest personal experience of adherence in the real-world setting (S3 Table).

#### Injection once every three months.

Both pregnant women and nurses/CHWs were concordant in their reasons for believing that the greatest positive effect on adherence would be afforded by a 3-monthly long-acting injectable due to the long duration of protection and the reduced burden of attending the clinic, including time and cost savings. Additionally, the personal preference of women for an injectable, and the dislike of many women for taking pills was highlighted. Pregnant women and nurses/CHWs who thought that a 3-monthly injection would impair adherence were concerned about patients’ phobia or dislike of injections. Although it was not suggested that ANC visits would be more infrequent, some pregnant women expressed the desire to come to the clinic more frequently than once every three months, indicating that they had assumed that ANC visits were dependent on the administration of chemoprevention. In contrast, one nurse expressed concerns regarding the increased workload while one CHW worried about the potential for women to forget to attend for the next injection because the doses were infrequent.

- *…3 months malaria prevention coverage is a welcome idea. That means I have fewer visits to the hospital, and this will afford me more time to do other activities. (Pregnant woman)*
- *It is cheaper and easier for them to take injections, and they do not have to visit the clinic every month because it covers them for a period of 3 months, saves them time, transportation and cost. (Nurse/CHW)*
- *Taking the tablets has been an issue. We see it on their faces when we want to administer the drugs. An injection that will last for three months will be much better. (Nurse/CHW)*
- *As I have difficulty swallowing the tablets, using an injectable form would be to my advantage in keeping to the course of treatment. (Pregnant woman)*
- *I do not like injections at all. In fact, I would prefer to take more tablets (the 4 tablets) than injections. (Pregnant woman)*
- *The duration between doses might be long enough to make them forget their next dose for the jab. (Nurse/CHW)*

#### Must be taken with food.

Both pregnant women and nurses/CHWs believed that having to take food before taking the drug reduced side effects and improved the absorption of the medicine and was therefore positive for adherence. Some women expressed a preference for eating before taking the drug and thought that food was needed for energy while pregnant. Nurses/CHWs expressed that food preferences and routines should not be changed during pregnancy, especially when women experience nausea, vomiting and appetite loss, though this was seen as both a positive and negative influence on adherence by this group, and only as a negative influence by pregnant women. There were also concerns from both stakeholder groups regarding the cost or access to food and that many women come to the clinic before they have eaten, so present with an empty stomach.

- *Because food promotes proper administration and absorption in the body, eliminating many of the undesirable effects associated with the drug. (Nurse)*
- *I’ll be tired from the effects of the medicine, I think with food I will have less side effects. (Pregnant woman)*
- *A lot of us pregnant women leave early for the facility without eating, as such it might be harder if there is an insistence for us to eat before taking the drug. (Pregnant woman)*
- *Most of the women will keep giving excuses of ‘I have not eaten’ and this will bring down compliance. (Nurse)*

#### IPTp delivery by CHWs.

Opinions on IPTp delivery by CHWs were also evenly split between those who thought it would improve and those who thought it would decrease adherence. Both pregnant women and nurses/CHWs agreed that the convenience of home visits enhanced adherence. Pregnant women also cited savings on transportation costs and removing the need to travel to the clinic, particularly if feeling unwell or tired, as positive factors. Nurses/CHWs highlighted that CHWs were members of their community and had a good relationship and bedside manner with patients. Both pregnant women and nurses/CHWs thought that the key negative perceptions of CHW IPTp delivery were their insufficient knowledge and capabilities and a distrust of CHWs in comparison to the higher levels of trust afforded to clinic staff. Nurses/CHWs also thought that CHWs would need to ensure DOT to support adherence, whereas there is better adherence monitoring at the clinic.

- *When I consider issues such as barriers to accessing hospital like distance, lack of transport fare… then it becomes easy for me when it is brought to my home by CHWs. (Pregnant woman)*
- *They will reach targeted recipients because of their knowledge of community members including their health and social status. (Nurse/CHW)*
- *Because I don’t trust the community agents, as they are poorly qualified and exposed to outside manipulation. (Pregnant woman)*
- *The CHWs are not competent enough in terms of clinical issues that may arise and how to handle the pregnant mothers. Documentation will be an issue. (Nurse/CHW)*
– *Because at the clinic, my condition is well monitored. The weight is well determined, the dosage of the medication is well explained and drug interactions are taken into account. (Pregnant woman)*

#### Some tablets taken at home.

Views around taking some tablets at home were equivocal among pregnant women. Some thought it would improve adherence, highlighting the importance of chemoprevention, the need to follow the healthcare provider’s instructions and the comfort of taking the medicine at home where side effects are more manageable. In contrast, those that expressed doubts had concerns regarding underdosing if women forget to take their medicine, the lack of adherence monitoring by healthcare workers and their dislike of taking drugs which may lead them to avoid doses if not directly observed at the clinic. Nurses/CHWs generally believed that taking medication at home would impair adherence, with concerns regarding underdosing, patients’ dislike of taking drugs and their experience of finding DOT challenging with some patients. However, some pregnant women and nurses/CHWs believed that adherence would remain unchanged as long as there was supportive patient education and clear instructions from healthcare providers.

- *I feel dizzy when I take drugs, so I can just take a part (which I think will reduce the effect) and then take the rest when I am home. It is safer if I’m home, so I can rest. (Pregnant woman)*
- *I still think it will remain the same as long as they are well educated about the need to take. (Nurse/CHW)*
- *A lot of us are taking it because they force us to take it in the clinic. There will be nobody to compel us at home to take it. (Pregnant woman)*
- *We usually struggle with DOT hence we can’t fully guarantee that they can take the tablets at home independently. (Nurse/CHW)*

#### Four tablets per day.

Most pregnant women and nurses/CHWs expected that four tablets per day would make adherence slightly/much harder offering reasons centered around the general dislike of taking drugs, the increased pill burden and concerns regarding underdosing if they could not complete the course. Where no effect or a positive effect on adherence was anticipated, it was because the number of tablets was considered secondary to following the prescriber’s instructions, and this approach could be supported through patient education and awareness programs.

- *I don’t like medications in the first place. It will be hard for me to take four tablets. (Pregnant woman)*
- *They don’t like the three tablets they are taking now, so if we increase it to four, most of them won’t take it and some will stop coming to the clinic. (Nurse/CHW)*
- *With proper counseling that we usually do I think they will just comply as usual. (Nurse/CHW)*
- *Knowing the importance attached to the medicine one has to take the drugs even though I do not much like swallowing tablets I will adjust. (Pregnant woman)*

### Community-based IPTp and new chemoprevention medicines

#### Perceptions of pregnant women on c-IPTp for new medicines.

**Fig 3:**
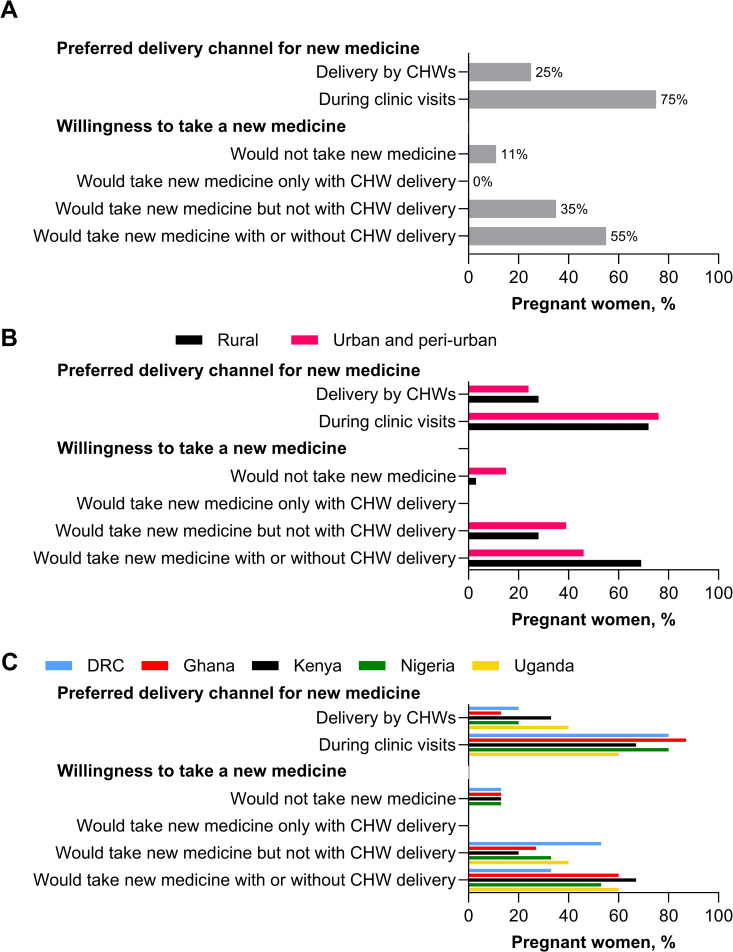
Preferences of pregnant women for delivery of new medicine and acceptance. Pregnant women were asked about their willingness to take a new medication that is identical to SP except that it is safe to use in the first trimester. They were then asked whether they preferred to receive the new medicine during clinic visits or via CHWs. **A)** All countries (n = 75). **B)** By setting (rural: n = 29; urban & peri-urban: n = 46). **C)** By country (Democratic Republic of Congo [DRC]: n = 15; Ghana: n = 15; Kenya: n = 15; Nigeria: n = 15; Uganda: n = 15). CHW, community health worker.

To gauge acceptance for a new chemoprevention therapy and to assess their preferences for delivery channel, either via ANC visits or CHWs, pregnant women were asked about their willingness to take a new medication that was identical to SP but safe to take in the first trimester. Three-quarters of pregnant women preferred delivery via the ANC visit, and none would accept a new medicine if delivered only by CHWs (Fig 3A). Women in rural areas appeared more open to CHW delivery and accepting of a new drug in general (Fig 3B). There were some differences in preference across countries, with CHW delivery most accepted in Uganda and Kenya, and least desired in Nigeria and Ghana (Fig 3C).

Further thematic exploration of these responses indicated that those who accepted a new medicine which was safe to start in the first trimester appreciated the longer protection from malaria but also needed to trust their healthcare workers that the intervention was safe (S4 Table). In contrast, those who did not accept this a new medicine with this profile were concerned about having to take more drugs for longer and had worries regarding the effects on the fetus and the ability to take medicine during early pregnancy.

- *At least I start getting protected from malaria from day one, that is enough reason to consider it. (Pregnant woman, Nigeria)*
- *I must take this medicine properly during all pregnancy to avoid malaria for me and my baby. (Pregnant woman, DRC)*
- *This is because I feel we are prone to miscarriage in the beginning of pregnancy as such I won’t want to take a medicine at this point. I also feel very weak in the first trimester and find it difficult to swallow. (Pregnant woman, Ghana)*

In terms of the preference for CHW versus ANC deployment for new chemoprevention medicines, pregnant women who favored administration at ANC visits appreciated the better instructions provided at the clinic, the wider range of services available, and had established trust with the clinic staff. Of the 55% (41/75) of pregnant women who were open to taking a drug from the first trimester when delivered by CHWs, most cited reasons of trust, issues with the availability and cost of transportation and the convenience of home visits. The main reasons for rejecting CHW delivery of a new medicine were associated with a distrust of CHWs versus a trust of clinic staff and the belief that CHWs have insufficient knowledge and skills. Similar responses were obtained when pregnant women were asked what they liked or disliked about CHW delivery. Positive opinions of CHW delivery were shared by 74 women, the most common being availability and cost of transport no longer being an issue, good bedside manner among CHWs with patients and the convenience of CHWs coming to patients’ homes. However, there were some country differences with the most common being good bedside manner in DRC, convenience of the CHW coming to the patients’ homes in Ghana and availability and cost of transport no longer being an issue in Kenya and Nigeria. These opinions were similar between urban/peri-urban and rural women. However, in Uganda, urban/peri-urban women cited the reduced burden of coming to hospital as the main advantage of CHW delivery as whereas rural women appreciated the availability and cost of transport no longer being an issue. Negative aspects were shared by just 32 respondents, with the most common being insufficient knowledge, distrust of CHWs by patients and the wider range of services at the clinic compared to CHWs.

- *Since they [CHWs] are professionals, I believe the drug is safe for the health of myself and my baby. It will save me from the cost of transportation (Pregnant woman, Ghana)*
- *They [CHWs] are always at our door step, so it’s very convenient for us. (Pregnant woman, Ghana)*
- *Any drug administered outside the clinic, I am not too comfortable with it. The doctors and nurses in my opinion are well trained compared to community health workers. (Pregnant woman, Nigeria)*
- *I will miss out on possible examinations that are done in a clinical set up. (Pregnant woman, Uganda)*
- *I don’t like anything about their services, they [CHWs] are not medics and I don’t trust them. (Pregnant woman, Kenya)*

#### Perceptions of CHWs on c-IPTp for new medicines.

Of the 15 CHWs surveyed, nine thought that it was a good idea for them to distribute IPTp with a new chemoprevention product, with the main reasons being protection against malaria, the convenience of home visits for pregnant women and that patients know and trust CHWs. However, only in DRC (3/3) and Uganda (3/3) did all CHWs think that they should deliver a new drug, followed by Nigeria (2/3) and Kenya (1/3), but with no support in Ghana (0/3). Reservations expressed in Ghana, Kenya and Nigeria included the need for pregnant women to attend ANC check-ups, worries about patients thinking that drugs were fake and the need for support and sufficient resources.

- *I will feel better because like the immunization program, we go from house to house to deliver. It is to save the child and the mother. (CHW, Ghana)*
- *I feel it will be okay if the staff strength will be enough, but going by our [CHW] number, it might not be enough. Some pregnant women might be left out. (CHW, Ghana)*
- *I don’t feel OK about it. Some patients might not fancy it as they think they might be fake drugs. (CHW, Nigeria)*
- *I don’t advocate for that since pregnant women are quite sensitive, hence they need check-up in a clinical set-up. (CHW, Kenya)*

#### Clinicians and policymakers: general views on c-IPTp.

Overall, policymakers and clinicians estimated that, based on their experience, delivering IPTp within the community setting would have a positive effect on uptake. Thematic evaluation revealed that across all the countries, clinicians and policymakers thought that the main reason for CHW delivery increasing IPTp uptake was the convenience of home visits (S5 Table). In Kenya, Nigeria and Uganda, the availability and cost of transport was a key consideration, and in DRC, Ghana and Nigeria the consideration of CHWs as members of the community who could build rapport with patients was considered important. In Uganda and Ghana, CHWs were seen as a means of reaching women in remote areas. In DRC, it was thought that CHWs could overcome the barrier of long waiting times at the ANC clinic. However, even if CHWs were used to implement IPTp, some women would remain unreached, primarily because they may be unavailable or inaccessible through work or childcare commitments. In DRC, distrust of CHWs was thought to impair delivery by this route, and in Ghana, Kenya, Nigeria and Uganda, it was believed that some patients would be unconvinced to receive drugs from CHWs. Socio-cultural issues were also cited in all countries except Ghana, such as CHWs not spending enough time in their designated communities, discrimination against teenage pregnant women and around cultural, religious or traditional beliefs related to pregnancies. In Ghana, bad roads were highlighted as a key barrier to CHW drug delivery.

- *[CHWs] will cushion the mothers from some of the barriers of attending clinics like accessibility and transport costs. As at now we are at 90% of IPTp-1 (1*^st^ *dose) however, the major challenge is IPTp-2 (2*^nd^ *dose) onwards where the numbers keep on reducing. (Policymaker, Uganda)*
- *This is because we have most of our CHW in the rural areas and these are the communities that are very difficult to reach. Hence, about 50% can be reached. (Clinician, Ghana)*
- *Basically, for CHW, if the condition of service doesn’t favor them, they won’t be interested in going out to deliver their service. [...] The conditions provided for the health workers to deliver their services can also be a barrier – are there stipends provided for them? Will this treatment be the only thing they are delivering or is it going to be a package of care for pregnant women and children under 5? All these will serve as barriers to achieving a 100% of reach by CHW. (Policymaker, Nigeria)*

### Direct choice experiment of next-generation IPTp product profiles

**Fig 4:**
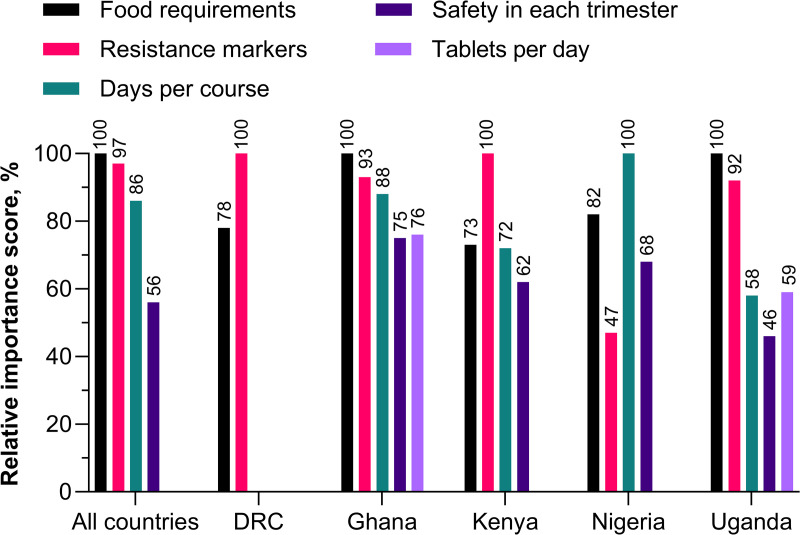
Discreet choice experiment findings for clinicians and policymakers. Product profile preferences were used to estimate the relative importance of product characteristics. Data for statistically significant characteristics are shown. Impact on birthweight was not statistically significant in any country. Example interpretation: for all countries, if food requirements is 100%, then resistance markers is 97% as important as food requirements, and days per course is 86% as important as food requirements, and safety in each trimester is 56% as important as food requirements. (all countries: n = 126; Democratic Republic of Congo [DRC]: n = 25; Ghana: n = 25; Kenya: n = 25; Nigeria: n = 26; Uganda: n = 25).

Different sample product profiles for new oral chemoprevention drugs were compared in the DCE by clinicians and policymakers. Analysis revealed that across all five countries, four of the six product characteristics were significant to product preference (Fig 4). Food requirements and resistance markers were the most influential factors in product selection, with days per course following closely behind. Safety in the first trimester exerted a moderate influence. Overall, tablets per day and effect on birthweight were not significant. However, tablets per day was a significant characteristic in Ghana and Uganda. Effect on birthweight was not a significant characteristic in any country. Resistance markers had a lower relative importance in Nigeria than other countries. Also, Nigeria was the only country where days per course was the most important attribute (Fig 4).

**Fig 5:**
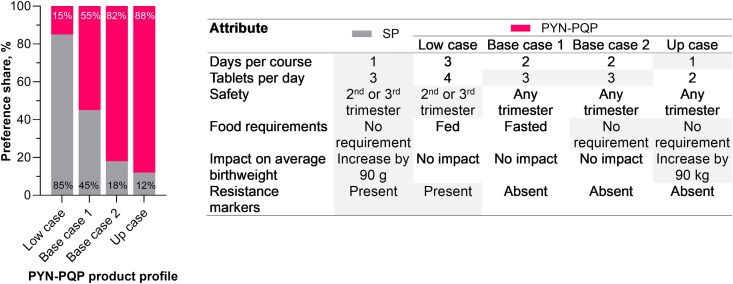
Effect on preference share of different pyronaridine-piperaquine product profiles. Preference share based on sulfadoxine-pyrimethamine (SP) versus four different potential pyronaridine-piperaquine (PYN-PQP) profiles.

These findings were used to estimate the preference share relative to SP for four most likely PYN-PQP product profiles out of the 216 profiles generated through the choice experiment (Fig 5). The preference share for PYN-PQP depended heavily on the specific characteristics, varying from 15% for the least favorable profile to 88% for the most favorable one (Fig 5). For example, changing the ‘base case’ profile from ‘requires fasting’ to ‘no food requirement’ increased relative PYN-PQP preference share from 55% to 82%, whereas the additional combined benefits of a single-day course of two tablets and an increase in birthweight of 90 g only boosted the PYN-PQP preference share from 82% to 88%.

## Discussion

This study aimed to characterize stakeholders’ preferences for potential next-generation IPTp product profiles, identifying their potential impact on acceptability and adherence as well as the potential for community-based delivery of IPTp with new medicines.

The aim of a new chemoprevention product is to protect as many women as possible from malaria for the full duration of their pregnancy. As malaria infection during the first trimester is associated with an increased risk of maternal anemia and low birthweight [38–40], suitability for use in the first trimester is a critical consideration, offering significant positive health outcomes. Nonetheless, this study highlights that any claims regarding use in first trimester must be supported by robust evidence, particularly in light of concerns about fetal fragility during this period. While developing a suitable drug for use in the first trimester is a significant advancement, its potential public health impact will remain constrained if early ANC attendance remains low or is delayed to later in pregnancy. Therefore, strengthening health systems to promote early ANC visits, including increasing awareness, reducing barriers to access and ensuring provider readiness, will be essential to maximize the benefits of expanded chemoprevention. Thus, there is the potential for a win-win scenario where the promotion of early ANC visits and availability of first-trimester chemoprevention could work in conjunction to improve maternal health and pregnancy outcomes.

Stakeholders across all groups agreed that transitioning from a monthly oral regimen to a 3-monthly long-acting injectable could significantly enhance adherence. This finding is supported by previous research in low- and middle-income countries where injectable therapies, such as long-acting contraceptives, HIV pre-exposure prophylaxis and tuberculosis treatments, have been associated with improved acceptability and adherence compared to oral regimens [41–45]. The potential benefits of an injectable chemopreventive therapy include extended protection against malaria, reduced overall pill burden in pregnancy, elimination of food restrictions and administration by DOT, ensuring women receive full protection without reliance on at-home compliance. However, challenges such as the need for trained personnel and potential logistical barriers to widespread implementation must be carefully considered. Additionally, we identified that some women thought that a 3-monthly chemotherapy regimen would reduce the availability of ANC visits, and there is also the risk that some women would skip ANC visits if they believed that receiving an injectable was the main purpose of ANC attendance. Should a long-acting injectable product become recommended, studies would need to evaluate the feasibility of integrating injectable IPTp products into routine ANC visits, how to differentiate between the need for ANC attendance and administration of an injectable malaria chemopreventive medicine and generate more evidence on its acceptability among pregnant women and healthcare providers.

In terms of oral chemoprevention, there was consensus that increasing days per course and/or tablets per course compared to SP would not be acceptable for many pregnant women. These concerns were mainly around adherence if women had to take tablets at home or had to swallow more tablets when three tablets is already quite challenging for some women. However, if there was confidence in the efficacy of a medicine with a longer regimen and/or greater pill burden, supported by patient education, the negative impact on adherence could potentially be overcome.

Issues around food requirements were also linked to adherence. To maximize adherence, pregnant women, CHWs and nurses highlighted that food could help mitigate adverse effects. This aligns with previous studies demonstrating that adverse events are increased following SP intake when pregnant women have not eaten prior to taking their medication, which in turn compromises adherence, with women refusing to take their medications on an empty stomach [46–52]. In previous studies investigating DHA-PQP for IPTp, the fasting requirement was identified as a barrier to adherence [49], and women’s preference for food intake was noted [53], as in the current study. Conversely, there were concerns among pregnant women regarding the cost and availability of food and women often arrive at the clinic without having eaten. In this case, a requirement for food before medication would limit adherence. A formulation that permits administration with or without food could offer clinical advantages by accommodating variability in food availability and individual preferences. This flexibility enables women to manage side effects according to their personal experiences, potentially improving efficacy and adherence to treatment [8,54]. Therefore, as future IPTp products are developed, minimizing the number of tablets and the number of days for the dosing regimen and eliminating food-related restrictions would all support adherence [44].

Among clinicians and policymakers, drug resistance was the second most influential factor in IPTp product preference, reflecting concerns about the long-term sustainability of chemoprevention. For the recombination of PYN with PQP, using components that are also indicated in malaria treatment regimens could potentially increase the risk of resistance selection, compromising the efficacy of current first-line treatment options. Policy considerations also highlight the need to reserve ACTs exclusively for malaria treatment [5]. The introduction of new alternatives for IPTp will require robust resistance surveillance, validation of molecular markers relevant to African parasites and efficacy monitoring.

Community-based IPTp delivery has been recommended by the WHO as a complementary approach to ANC-based services and has been shown to increase IPTp3 + uptake in several countries [5,7,55]. The intervention has been piloted in DRC, Madagascar, Mozambique and Nigeria as part of the TIPTOP project [35], and in separate projects in Burkina Faso, Malawi, Sierra Leone and Senegal [56]. Our findings suggest that CHW-led IPTp distribution could reach more women, particularly those facing geographic or socio-economic barriers to ANC access. However, in terms of introduction of new chemoprevention medicines, stakeholder perspectives on c-IPTp were mixed. While most CHWs and policymakers believed that CHW delivery could improve adherence, pregnant women expressed a strong preference for receiving IPTp at ANC clinics, citing greater trust in facility-based providers and concerns about CHW-administered medications. Despite the experience of TIPTOP in DRC and Nigeria, women’s openness to receiving a new medicine via CHWs was muted, with most women in these countries preferring ANC delivery. In DRC in particular, 53% of women would take a new drug if it were not via CHW delivery, versus only 33% if it were with or without CHW delivery. Women in Kenya and Uganda appeared most open to a new chemoprevention medicine delivered via CHWs even though the intervention has not been extensively piloted in these countries. Thus, it cannot be assumed that the acceptance of community-based delivery of IPTp with SP could automatically be transferred to a new and unfamiliar chemoprevention medicine.

Importantly, none of the pregnant women surveyed were willing to accept a new IPTp drug exclusively distributed by CHWs. This aligns with prior research showing that trust in CHWs varies across contexts and that socio-cultural barriers, such as decision-making process at household level, peer influence and limited health literacy, can impact uptake [8,57–63]. Similarly, CHWs have reported the importance of supportive supervision and close connection with professional healthcare workers [64]. Additionally, aligning with the social norms around pregnancy [65], such as involving female CHWs and linking activities to national health programs, can enhance legitimacy [57]. To maximize the impact of c-IPTp, implementation strategies should focus on strengthening CHW training and supervision, fostering community trust and integrating CHWs more closely with formal healthcare structures [57, 64, 65]. Finally, targeted education campaigns could help improve awareness of IPTp benefits and mitigate concerns about CHW-administered interventions. However, based on the findings of this study, it is likely that a new medicine will need to be distributed via ANC visits, at least in the first instance, though also possibly via CHWs once trust has been established.

The DCE was applied to further assess clinician and policymaker preferences. To our knowledge, this is the first time that a DCE instrument has been applied to investigate a malaria chemoprevention product. This research design employs an efficient methodology to identify stakeholders’ priorities to guide the development of next-generation IPTp products. Notably, the DCE and survey findings cannot be directly compared. This is because the DCE presents a closed choice of preferred characteristics between product profiles, while the survey explored opinions on key issues which can be positive or negative. As expected, the two approaches gave different results, but the application of the findings is complementary. The DCE is valuable in assessing the potential market penetration of a new product against an existing offering; it is specific and narrow in its application. In contrast, the survey provides more nuanced information on acceptability of chemoprevention products across diverse stakeholders but is more generalized, allowing exploration of the reasons underlying stakeholder perceptions. For example, the DCE findings highlighted the importance of food requirements, with policymakers and clinicians preferring product profiles without food restrictions. The survey results indicated that this was mainly because of the impact of food on adherence. Similarly, markers of resistance was also considered a key consideration in the DCE, with the survey revealing that this centered around both addressing SP resistance and ensuring that any new medicine was effective at preventing malaria. There was notable heterogeneity across countries in the DCE. In Nigeria, markers of resistance were about half as important as days per course. Tablets per day was only a significant consideration in Ghana and Uganda. However, any new medicine will have to meet requirements across all countries. In summary, the DCE suggests a new medicine must have no food requirement or markers of resistance and have days per course and tablets per day similar to SP. However, an improvement in birthweight similar to that observed with SP would not be a critical factor in choosing a new medicine over SP. Thus, convenience factors that drive adherence and efficacy are the key considerations for product development.

A key limitation of the study is the use of a convenience sample which may have led to selection bias despite mitigations. Also, the relatively small sample size for each stakeholder group in each country restricts the interpretation of country differences. Qualitative investigations were not done to achieve thematic saturation, because the aim was to identify only the key responses within each theme. In addition, not all regions and districts were represented due to the size of the sample and caution should be used when generalizing the results of this subset of respondents to the entire malaria-endemic region of sub-Saharan Africa. To mitigate the impact of the small sample size we carefully selected participants who were the most knowledgeable for this survey. We amalgamated responses into four levels, pregnant women, nurses/CHWs, clinicians and policyholders based on their service access or provision. However, there is likely to be some diversity within aggregated stakeholder groups, for example, nurses and CHWs have different patient-facing roles; also, their roles may differ among countries. More in-depth studies with larger sample sizes would be needed to dissect the differences among CHWs and nurses and between countries, which may be significant in terms of c-IPTp implementation. However, the sample size and methods were considered sufficient to inform the aims of the study in terms of evaluating stakeholder preferences relevant to decision making in the development of new chemoprevention medicines. As with any survey, our findings reflect the answers and views of participants that may be influenced by their recall and response bias. Notably, as we compared an established product (SP) with a hypothetical one (PYN-PQP), participants might have been biased by their experience with SP. To mitigate this risk, clear guidance was provided to interviewers to ensure that they obtained the most accurate information. Strengths of this study are the combination of quantitative open-ended data gathering and analysis, the focused research framework, and the diversity of respondents. More specifically, few studies include the whole range of stakeholders, from decision makers to end-users. This approach allowed direct comparison of responses and identified consensus and discrepancies amongst target groups. Additionally, the DCE was shown to be an efficient method for evaluating antimalarial product profiles, translating into preference choices to inform drug development goals. Such an approach could be used for any new antimalarial chemoprevention product and could also be applied to new malaria treatments. Importantly, this study is notable for engaging stakeholders early in the development process for new medicines.

## Conclusions

These findings offer important considerations for the development and deployment of next generation of oral and injectable products for malaria chemoprevention during pregnancy. Expansion of chemoprevention into the first trimester of pregnancy would increase protection, but the benefit/risk must be thoroughly defined. Adherence is a key consideration, and factors which support adherence are priorities, including minimizing food-related restrictions and dosing regimens which did not increase pill burden or days per course. In addition, enhanced resistance monitoring and further research into the resistance mechanisms of novel chemoprevention agents will be needed to support adoption and long-term efficacy. A key finding was that long-acting injectable formulations have the potential to transform IPTp protection duration and adherence. However, further research is needed to identify and address potential implementation challenges. Community-based IPTp delivery could extend coverage to unreached populations, but especially for new medicines, concerns around trust and acceptability must be addressed through targeted engagement and education strategies. Overall, the findings suggest that for the introduction of new oral chemoprevention products, expanding IPTp reach while respecting women’s preferences for ANC-based care, will likely require a hybrid delivery model combining IPTp via both ANC visits and CHW delivery. However, the most appropriate mix will be context and product specific and should be supported by implementation research. This requires continuous engagement with the community through participatory qualitative research to ensure pregnant women’s perceptions are well captured, understood and their needs properly addressed.

## Supporting information

S1 Table Participant characteristics by country. (DOCX)

S2 Table Qualitative evaluation of the importance of chemoprevention product attributes. (DOCX)

S3 Table Qualitative evaluation of pregnant women’s and nurses/CHWs’ responses on factors influencing adherence. (DOCX)

S4 Table Willingness of pregnant women to take a new chemoprevention medication and preferred delivery channel. (DOCX)

S5 Table Reasons for the improved reach of chemoprevention with CHW delivery: clinicians and policymaker perspectives. (DOCX)
