# Supplementary material for: Improving malaria chemoprevention coverage in pregnancy: Surveying stakeholder preferences for new product profiles and community-delivery approaches across five African countries
Source: PLOS Glob Public Health. 2026 Mar 13;6(3):e0005607. doi: 10.1371/journal.pgph.0005607 (PMC12987456; doi:10.1371/journal.pgph.0005607)
Supplement: S1 Table — (DOCX) [file pgph.0005607.s001.docx]

# S1 Table. Participant characteristics by country.

| Category | Characteristic | | All | | DRC (n=53) | | Ghana (n=50)_ | | Kenya (n=50) | | Nigeria (n=51) | | Uganda (n=50) | |
| --- | --- | --- | --- | --- | --- | --- | --- | --- | --- | --- | --- | --- | --- | --- |
|  |  |  | Percent | N | Percent | N | Percent | N | Percent | N | Percent | N | Percent | N |
| All | Location of interview | | -- | -- | Kinshasa: 60%  Kenge: 23%  Boma: 17% | 32  12  9 | Accra: 30%  Tamale: 18%  Bekwai: 8%  Kumasi: 8%  Obuasi: 8%  Savelugu: 8%  Yendi: 6%  Kanvilli: 4%  Tema: 3%  Vittin: 2%  Bakwai: 1% | 15  9  4  4  4  4  3  2  2  2  1 | Kilifi: 20%  Kisumu: 16%  Nairobi: 16%  Mombasa: 12%  Bungoma: 6%  Kakamega: 6%  Voi: 6%  Bondo: 4%  Busia: 4%  Mwatate: 4%  Homabay: 2%  Malindi: 2%  Siaya: 2% | 10  8  8  6  3  3  3  2  2  2  1  1  1 | Abuja: 31%  Lagos: 29%  Port Harcourt: 18%  Kano: 16%  Benin: 6% | 16  15  9  8  3 | Iganga: 36%  Kampala: 34%  Jinja: 30% | 18  17  15 |
| Pregnant women | Location of home | Rural  Peri-urban  Urban | 39%  27%  35% | 75 | 13%  40%  47% | 15 | 53%  20%  27% | 15 | 47%  33%  20% | 15 | 40%  27%  33% | 15 | 40%  13%  47% | 15 |
|  | Currently pregnant | | 80% | 75 | 87% | 15 | 80% | 15 | 80% | 15 | 67% | 15 | 87% | 15 |
|  | Attended at least one ANC visit | | 100% | 75 | 100% | 15 | 100% | 15 | 100% | 15 | 100% | 15 | 100% | 15 |
|  | Took at least one dose of IPTp-SP | | 95% | 75 | 87% | 15 | 87% | 15 | 100% | 15 | 100% | 15 | 100% | 15 |
|  | On cotrimoxazole | | 0% | 75 | 0% | 15 | 0% | 15 | 0% | 15 | 0% | 15 | 0% | 15 |
| Clinicians, nurses and CHWs | Location of work | Rural  Peri-urban  Urban | 27%  33%  40% | 128 | 18%  32%  50% | 28 | 36%  40%  24% | 25 | 32%  36%  32% | 25 | 32%  36%  32% | 25 | 20%  20%  60% | 25 |
|  | Sector of direct care | Public/ government  Private/NGO/  faith-based | 69%  31% | 108 | 41%  59% | 27 | 80%  20% | 20 | 74%  26% | 19 | 77%  23% | 22 | 80%  20% | 20 |
|  | Provide direct care to pregnant women | | 94% | 115 | 100% | 27 | 91% | 22 | 86% | 22 | 100% | 22 | 91% | 22 |
| Policy makers | Years in current role, median (IQR) | | 5.0  (2.2–8.5) | 51 | 7.0  (6.2–9.5) | 10 | 4.0  (1.2–5.0) | 10 | 5.0  (1.0–10.0) | 10 | 3.0  (2.8–4.5) | 11 | 6.0  (3.5–8.8) | 10 |
|  | Years working in field of malaria, median (IQR) | | 14.0  (8.5–22.0) | 51 | 22.5  (18.8–27.2) | 10 | 14.0  (10.8–20.5) | 10 | 10.0  (5.8–18.8) | 10 | 10.0  (8.5–17.0) | 11 | 15.0  (8.2–17.5) | 10 |
|  | Level of authority for decisions about a new antimalarial | Low  Medium  High | 6%  47%  47% | 51 | 0%  40%  60% | 10 | 0%  80%  20% | 10 | 10%  30%  60% | 10 | 18%  36%  45% | 11 | 0%  50%  50% | 10 |

ANC, ante-natal clinic; CHW, community health worker; IPTp, intermittent preventive treatment in pregnancy; IQR, interquartile range; NGO, non-governmental organization.
