# Supplementary material for: Improving malaria chemoprevention coverage in pregnancy: Surveying stakeholder preferences for new product profiles and community-delivery approaches across five African countries
Source: PLOS Glob Public Health. 2026 Mar 13;6(3):e0005607. doi: 10.1371/journal.pgph.0005607 (PMC12987456; doi:10.1371/journal.pgph.0005607)
Supplement: S2 Table — (DOCX) [file pgph.0005607.s002.docx]

# S2 Table. Qualitative evaluation of the importance of chemoprevention product attributes.

| **Attribute** | **High/very high importance** | **Medium importance** | **Low/very low importance** |
| --- | --- | --- | --- |
| Safety | - Want a drug that is safe to take in any trimester (174/232) - Protection from malaria (general response) and/or its complications/severity of illness for mother and/or baby (^61/232) - Drugs have a teratogenic effect that can affect the period of organogenesis (30/232) | - Want a drug that is safe to take in any trimester (6/18) - Drugs have a teratogenic effect that can affect the period of organogenesis (5/18) - Secondary importance (4/18), with no specifics on what parameters or factors are more important | - Want a drug that is safe to take in any trimester (1/4) - Drugs have a teratogenic effect that can affect the period of organogenesis (1/4) - Safety is of secondary importance (1/4), with no specifics on what parameters or factors are more important except that patients should be adherent |
| Resistance markers | - Protection from malaria (general response) and/or its complications/severity of illness for mother and/or baby (^86/106) - Concern about drug resistance (12/106) - Find an alternate medicine/molecule (4/106) | - Secondary importance (5/14) with other reasons being more important such as ‘other characteristics’ or benefits like impact on birthweight - Protection from malaria, general response (3/14) - Preventative medicine (2/14) | - Protection from malaria (general response) and/or its complications/severity of illness for mother and/or baby (^6/6) - Not concerned about drug resistance (2/6) - Additional research is needed on the resistance (1/6) |
| Days per course | - Compliance and adherence to the treatment plan (75/206) - Concerns over underdosing (not finishing the course), such as forgetting to take the drug and/or aspects of pregnancy affecting uptake (^65/206) - Patient’s phobia or dislike of drugs (32/206) | - Secondary importance (19/36), with other reasons given as more important such as efficacy, but also calling attention to patient education | - Secondary importance (12/12), with other reasons being more critical such as efficacy, protection from malaria and its complications for mother and/or baby, and the patient’s ability to tolerate the drug |
| Tablets per day | - Pill burden, i.e., taking too many tablets (58/196) - Concerns over underdosing (not finishing the course), such as forgetting to take the drug and/or aspects of pregnancy affecting uptake (^52/196) - Patient’s phobia or dislike of drugs (42/196) | - Secondary importance (16/46), with other reasons given as more important such as ensuring patient education to ensure compliance and protection from malaria and its complications for mother and/or baby - Patients have a personal preference (or appeal) for the number of tablets they are comfortable taking (7/46) | - Secondary importance (9/12), with multiple other reasons being more important such as efficacy, on-site monitoring, and number of days taking precedence |
| Food requirements | - Food preferences and routines, and/or changes in food routines during pregnancy when they experience nausea/vomiting/ appetite loss (^37/137) - Food reduces the side effects caused by the drug (34/137) - Cost or access to food (18/137) | - Food preferences and routines, and/or changes in food routines during pregnancy when they experience nausea/vomiting/ appetite loss (^27/77) - Cost or access to food (10/77) - Secondary importance (9/77) – with other varied reasons given more importance, such as efficacy and safety | - Secondary importance (12/40), with other varied reasons given more importance, such as efficacy, patient sensitization, and food requirements under DOT - Food requirements should still ensure compliance or adherence to the plan (6/40) - Food preferences and routines, and/or changes in food routines during pregnancy when they experience nausea/vomiting/ appetite loss (^5/40) |
| Impact on birthweight | - Big or heavy babies are considered healthy babies (23/49) - Helps low birthweight babies (11/49) - Mothers like big babies (8/49) | - 90g is an insignificant amount of weight gain for the baby (11/15) - Secondary importance (4/15), with other reasons as more important such as protection against malaria | - Difficult delivery (5/11) - Mother does not want big baby (4/11) |

Participants ranked the importance of attributes on a 5-point Likert scale (very high, high, medium, low, very low) and were then asked to qualify their responses. The top three reasons for preferring a product attribute are presented, based on the number of times a respondent stated this reason (numerator), within a respondent category (denominator). ^ indicates the frequency count of nuanced reasons that are related to each other.

DOT, directly observed therapy.
