# Supplementary material for: Improving malaria chemoprevention coverage in pregnancy: Surveying stakeholder preferences for new product profiles and community-delivery approaches across five African countries
Source: PLOS Glob Public Health. 2026 Mar 13;6(3):e0005607. doi: 10.1371/journal.pgph.0005607 (PMC12987456; doi:10.1371/journal.pgph.0005607)
Supplement: S3 Table — (DOCX) [file pgph.0005607.s003.docx]

# S3 Table. Qualitative evaluation of pregnant women’s and nurses/CHWs’ responses on factors influencing adherence.

| **Factor** | **Category** | **Much/slightly easier or Much/slightly higher** | **Neither harder nor easier/ the same** | **Slightly/much harder or Slightly/much lower** |
| --- | --- | --- | --- | --- |
| Injection once every 3 months | Pregnant women | - Duration of protection (injection given once every three months (30/48), hence reducing the burden of coming to the hospital, including time and cost savings (^12/48) - Personal preference or appeal (17/48) - Phobia or dislike of drugs (6/48) | - Duration of protection (injection given once every three months (2/6) - Personal preference or appeal (including being able to visit the clinic for better healthcare and services) (2/6) | - Patient’s phobia or dislike of injections (12/21) - Want to come to the clinic more frequently than once every three months since duration of protection is now longer (3/21) |
|  | Nurses/CHWs | - Duration of protection (injection given once every three months (28/39), hence reducing the burden of coming to the hospital, including time and cost savings (^10/39)) - Personal preference or appeal (16/39) - Patient’s phobia or dislike of drugs (7/39) | - Patient’s phobia or dislike of injections (4/6) - Duration of protection (injection given once every three months (2/6)) - Patient’s phobia or dislike of drugs (1/6) | - Patient’s phobia or dislike of injections (7/8) - Adds burden on the hospital/clinic staff (1/8) - Forget to take the next injection because doses are so far apart (1/8) |
| Must be taken with food | Pregnant women | - Personal preference to eat before taking medicine (11/38) - Food reduces the side effects caused by the drug (6/38) - Food provides energy (6/38) - Food contributes to the absorption of medicine (5/38) | - Secondary importance (5/18) -- with other reasons being more important such as following doctors’ instructions and protection from malaria and its complications for mom and/or baby - Food preferences and routines, and/or changes in food routines during pregnancy when they experience nausea/vomiting/ appetite loss (^4/18) | - Food preferences and routines, and/or changes in food routines during pregnancy when they experience nausea/vomiting/ appetite loss, thus negatively affecting their ability to take the medicine (^10/19) - Patients come on an empty stomach (3/19) - Cost or access to food (2/15) |
|  | Nurses/CHWs | - Food reduces the side effects caused by the drug (6/14) - Food contributes to the absorption of medicine (4/14) - Food preferences and routines, and/or changes in food routines during pregnancy when they experience nausea/ vomiting/ appetite loss (^4/14) | - Food preferences and routines, and/or changes in food routines during pregnancy when they experience nausea/vomiting/ appetite loss (^6/13) - Efficacy of drug (3/13) - Existing patient education (2/13) | - Food preferences and routines, and/or changes in food routines during pregnancy when they experience nausea/vomiting/ appetite loss, that negatively affect the ability to take the medicine (^17/26) - Cost or access to food (7/26) - Patient comes on an empty stomach to the clinic (6/26) |
| Delivery by CHWs | Pregnant women | - Save on transportation costs (13/39) - Convenience of CHWs coming to your home to provide the drug (12/39) - Removing the burden of coming to the clinic (8/39) | - Secondary importance (4/11) – with other varied reasons being more important such as patients will still go to the clinic for antenatal care or to check with the doctor, and protection from malaria and its complications for mom and/or baby - Wider range of services available at the hospital (3/11) | - Belief that CHWs have insufficient knowledge and capabilities to provide such a drug (6/25) - Distrust of CHWs (6/25) - Trust clinic staff and would like the medicine from these type of healthcare workers (5/25) |
|  | Nurses/CHWs | - Convenience of CHWs coming to your home to provide the drug (8/21) - CHWs are members of the community (6/21) - Good bedside manner with patients (5/21) | - Secondary importance (3/6) - with varied reasons, such as CHWs can conduct DOT to ensure compliance, patient sensitization, etc. - CHW professionalism (2/6) | - Belief that CHWs have insufficient knowledge and capabilities to provide such a drug (6/26) - Distrust of CHWs (5/26) - Requires CHWs to monitor patients to take drugs in front of them to ensure patients are compliant and/or better monitoring of patients at the clinic (^5/26) |
| Some tablets taken at home | Pregnant women | - Protection from malaria (general response) and/or its complications/severity of illness for mom and/or baby (^14/28) - Comfort of taking at home due to side effects of the drug on the patient or ability to rest (5/28) - Secondary importance (3/28) -- with other reasons being more important such as follow doctors’ and nurses/CHWs’ instructions (3/3) | - Secondary importance (5/16) - with other reasons being more important such as following doctors’ instructions and protection from the complications of malaria - Concerns over underdosing (not finishing the course) such as forgetting to take the drug(4/16) - Do not have a problem taking the drugs at home (4/16) | - Concerns over underdosing (not finishing the course), such as forgetting to take the drug and/or aspects of pregnancy affecting uptake (^24/31) - Healthcare worker cannot monitor patient’s intake of tablets (9/31) - Patient’s phobia or dislike of drugs (5/31) |
|  | Nurses/CHWs | - Concerns over underdosing (not finishing the course) such as forgetting to take the drug (2/6) - Comfort of taking at home due to side effects of the drug on the patient or the ability to rest (2/6) | - Secondary importance (3/5) - with other reasons being more important such as the need to sensitize the patient to adhere or comply with the treatment plan - Patient’s phobia or dislike of drugs (2/5) | - Concerns over underdosing (not finishing the course), such as forgetting to take the drug and/or aspects of pregnancy affecting uptake (^46/42) - Patient’s phobia or dislike of drugs (7/42) - Even DOT is challenging with patients (5/42) |
| Four tablets per day | Pregnant women | - Extra tablet does not matter (2/8) - Protection from malaria (general response) and/or its complications/severity of illness for mom and/or baby (^2/8) - Days per course takes precedence (2/8) | - Secondary importance (8/15) - with other reasons being more important such as following doctors’ instructions and protection from the complications of malaria - Extra tablet does not matter (5/15) - Patient’s phobia or dislike of drugs (2/15) | - Patient’s phobia or dislike of drugs (25/52) - Pill burden, i.e., taking too many tablets (19/52) - Concerns over underdosing (not finishing the course), such as forgetting to take the drug (5/52) |
|  | Nurses/CHWs | - Concerns over underdosing (not finishing the course), such as forgetting to take the drug (6/8) - Pill burden, i.e., taking too many tablets (4/8) - Patient’s phobia or dislike of drugs (2/8) | - Patients are sensitized or counseled (patient education on the importance of the drug (2/13) - Secondary importance (3/13) - with other reasons being more important such as following doctors’ instructions, if patient sensitization is done to ensure compliance, and protection from malaria and its complications for mom and/or baby | - Pill burden, i.e., taking too many tablets (22/32) - Concerns over underdosing (not finishing the course), such as forgetting to take the drug (9/32) - Patient’s phobia or dislike of drugs (4/32) |

Pregnant women (n=75) and nurses/CHWs (n=53) were asked to qualify their answers attributes on a 5-point Likert scale considering factors which may affect adherence. The top three reasons for preferring a product attribute are presented, based on the number of times a respondent stated this reason (numerator), within a respondent category (denominator). ^ indicates the frequency count of nuanced reasons that are related to each other.

CHWs, community health workers.
