# Supplementary material for: Improving malaria chemoprevention coverage in pregnancy: Surveying stakeholder preferences for new product profiles and community-delivery approaches across five African countries
Source: PLOS Glob Public Health. 2026 Mar 13;6(3):e0005607. doi: 10.1371/journal.pgph.0005607 (PMC12987456; doi:10.1371/journal.pgph.0005607)
Supplement: S4 Table — (DOCX) [file pgph.0005607.s004.docx]

# S4 Table. Willingness of pregnant women to take a new chemoprevention medication and preferred delivery channel.

| **Question** | **Response** | |
| --- | --- | --- |
| Willingness to take a new chemoprevention drug identical to SP but safe to start in second trimester | YES (67/75; 89%)   - Want a medicine that is safe to start from the first trimester (54/67) - Protection from malaria (general response) and/or its complications/severity of illness for mom and/or baby (^15/67) - Secondary importance – with key reason being as long as it considered safe by healthcare workers like clinicians (8/67) | NO (8/75; 11%)   - Patient’s phobia or dislike of drugs (2/8) - First trimester is a delicate time (generic statement or discuss throwing up) (2/8) - Patient does not know what the effect will be (patient education) (1/8) |
| Willingness to take a new chemoprevention drug identical to SP but safe to start in second trimester and delivery by CHWs | YES (41/75; 55%)   - Trust CHWs (8/41) - Availability and cost of transport is no longer an issue (6/41) - Convenience of CHWs coming to your home to provide the drug (5/41) | NO (34/75; 45%);   - Distrust of CHWs (19/34) - CHWs have insufficient knowledge and skills (5/34) - Trust clinic staff (5/34) |
| Preference for delivery channel of the new medicine | During clinic visits (56/75; 75%)   - Better instructions or healthcare provided at the clinic (25/56) - Wider range of services available and provided at the clinic (so patients can be checked for other ailments or complications while at the clinic) (16/56) - Trust doctors and nurses at the clinic (15/56) | By CHWs (19/75; 25%)   - Availability and cost of transport is no longer an issue (14/19) - Long waiting times at the clinic/time savings (7/19) - The physical or mental state of the patient impacts the ability to go to the healthcare facility (5/19) |
| Likes and dislikes of CHW delivery | Likes (74/75 responded)   - Availability and cost of transport is no longer an issue (25/50) - Good bedside manner among CHWs with the patients (15/50) - Convenience of the CHW coming to the patients’ homes (12/50) | Dislikes (32/75 responded)   - CHWs have insufficient knowledge (6/32) - Distrust of CHWs by patients (6/32) - Patients can gain a wider range of services at the clinic compared to CHWs (5/32) |

Pregnant women (n=75) were asked on their willingness to take a new medicines identical to SP, except that it is safe to use in the first trimester and is delivered during clinic visits (Yes/No), and then asked which delivery mechanism they preferred, receiving the new medicine during clinic visits or via community health workers (CHWs). They were then asked to qualify their responses. The top three reasons for the responses are presented, based on the number of times a respondent stated this reason (numerator), within a respondent category (denominator). ^ indicates the frequency count of nuanced reasons that are related to each other.
