# Supplementary material for: Improving malaria chemoprevention coverage in pregnancy: Surveying stakeholder preferences for new product profiles and community-delivery approaches across five African countries
Source: PLOS Glob Public Health. 2026 Mar 13;6(3):e0005607. doi: 10.1371/journal.pgph.0005607 (PMC12987456; doi:10.1371/journal.pgph.0005607)
Supplement: S5 Table — (DOCX) [file pgph.0005607.s005.docx]

# S5 Table. Reasons for the improved reach of chemoprevention with CHW delivery: clinicians and policymaker perspectives.

| **Country** | **Why would some women move from unreached to reached with CHW delivery?** | **Why would some women remain unreached with CHW delivery?** |
| --- | --- | --- |
| DRC (n=25) | - Convenience of the CHW coming to the patients’ homes (7/19) - CHWs are members of the community (5/19) - Long waiting time at the clinic (3/19) | - Distrust of CHWs (10/25) - Patients are unavailable or inaccessible such as when CHWs come they may be tied in work or childcare (9/25) - Socio-cultural factors amongst the patients stand as barriers to receiving drugs from CHWs (4/25) |
| Ghana (n=25) | - Convenience of the CHW coming to the patients’ home (9/25) - CHWs can reach the patients including those in remote areas (9/25) - CHWs are members of the community (3/2) | - Patients are unavailable or inaccessible, such as when CHWs come, they may be tied in work or childcare (18/23) - Difficult patients who cannot be convinced to receive drugs from CHWs (6/23) - Bad roads impact CHWs’ ability to reach patients (6/23) |
| Kenya (n=25) | - Convenience of the CHW coming to the patients’ homes (9/24) - Availability and cost of transport is no longer an issue (9/24) - Burden of coming to the hospital is addressed (6/24) | - Patients are unavailable or inaccessible such as when CHWs come they may be tied in work or childcare (12/21) - Socio-cultural factors amongst the patients stand as barriers to receiving drugs from CHWs (6/21) - Difficult patients who cannot be convinced to receive drugs from CHWs (3/21) |
| Nigeria (n=26) | - Convenience of the CHW coming to the patients’ homes (12/24) - Availability and cost of transport is no longer an issue (9/24) - CHWs are members of the community (and can build rapport with the patients) (2/24) | - Socio-cultural factors amongst the patients stand as barriers to receiving drugs from CHWs (12/19) - Patients are unavailable or inaccessible such as when CHWs come they may be tied in work or childcare (7/19) - Difficult patients who cannot be convinced to receive drugs from CHWs (5/19) |
| Uganda (n=25) | - Convenience of the CHW coming to the patients’ homes (10/23) - Availability and cost of transport is no longer an issue (9/23) - CHWs can reach patients, including those in remote areas (4/23) | - Patients are unavailable or inaccessible such as when CHWs come they may be tied in work or childcare (12/25) - Socio-cultural factors amongst the patients stand as barriers to receiving drugs from CHWs (7/25) - Difficult patients who cannot be convinced to receive drugs from CHWs (6/25) |

The top three reasons for the responses are presented, based on the number of times a respondent stated this reason (numerator), within a respondent category (denominator). ^ indicates the frequency count of nuanced reasons that are related to each other. CHW, community health worker.
